# School environment factors were associated with BMI among adolescents in Xi'an City, China

**DOI:** 10.1186/1471-2458-11-792

**Published:** 2011-10-11

**Authors:** Ming Li, Michael J Dibley, Hong Yan

**Affiliations:** 1 Division of Health Sciences, University of South Australia, Australia; 2 College of Medicine, Xi'an Jiaotong University, Xi'an, China; 3 The School of Public Health, University of Sydney, Australia

## Abstract

**Background:**

School environment influences students' behaviours. The purpose of this research was to identify school environment factors associated with BMI.

**Methods:**

A cross-sectional study was conducted among 1792 school-aged adolescents from 30 schools in six districts in Xi'an City in 2004. Height and weight were taken from students by trained field staff. School environment characteristics such as physical factors (school facilities, school shops and fast food outlets in school area), school curricula and policies were collected from school doctors using school environment questionnaire. School environment factors were identified in linear mixed effect models with BMI as outcome and adjusted for socio-demographic factors.

**Results:**

After adjusted for socio-demographic factors, BMI was associated with the availability of soft drinks at school shops, the availability and the number of western food outlet in the school vicinity. School curricula such as sports-meeting and health education session were also associated with BMI.

**Conclusions:**

Urgent actions are needed to address the obesogenic elements of school environments. Community and school policy makers should make efforts for students to avoid exposure to fast food outlet in school area and soft drinks at school shops, and to improve school curricula to promote healthy behaviours.

## Background

Overweight and obesity among adolescents in China, especially in urban populations, has become a public health priority [1]. A survey in Xi'an City, a major urban area in northwest China with a population of 7 million, has revealed that 16% of the adolescents aged 11-17 were either overweight or obese based on the cutoffs of International Obesity Task Force (IOTF) [2].

Overweight and obesity are the result of positive energy balance. Although individual behavioural factors such as low levels of physical activity and/or dietary behaviours are thought to contribute to the epidemic of obesity, broader understanding of environmental factors has been extended. The Analysis Grid for Environments Linked to Obesity dissects the environment into micro or macro, in which the former includes factors related to school, workplaces, homes and neighbourhoods [3]. Davidson and Birth put forward a conceptual framework that suggests children's behaviour is embedded in the wide environment at community, school, and family levels [4]. Schools exert a great influence on children's behaviour since students spend most of their waking time during a day at schools (typically 8-12 hours per day). School time is the key developmental period in which children adopt health behaviours as lifetime habits.

A Cochrane review of obesity intervention studies undertaken in Western countries has shown that behavioural change interventions alone in adolescents had limited impact, either in the short-term or longer-term [5]. A systematic review of interventions in China among school students revealed that strategies focusing on health knowledge, physical activity and diet had limited beneficial effect for healthy weight [6]. These findings suggest that future obesity prevention programs should include changing school environments to facilitate change in individual behaviours and thus improve the impact and sustainability of obesity prevention programs [5].

Evidence of association between school environment factors and obesity was limited to recent studies on fast-food restaurant and retailers in school vicinity [7-9]. Other factors such as availability of sports facilities, school curriculum and policies with weight status and behaviours have been rarely studied [10-12]. The purpose of this study was to identify the school environment factors associated with BMI among school adolescent in Xi'an City to provide evidence for future research and prevention programs in adolescents in urban China.

## Methods

From May to November in 2004, a sample of 1804 adolescents aged 11-17 years representing junior high students in Xi'an City were enrolled in this survey. The mean age of the adolescents was 13.9 years. There were 49.8% females and 50.2% male students. A multistage cluster sampling method was used in which 30 out of 183 junior high schools were selected proportionate to school population size (ranging from 258 to 3744 students). In each selected school, one class from each grade was randomly selected and from each class 20 students were selected using systematic random sampling.

Informed signed consent was sought hierarchically from the Municipal Education Department, the school health division at district level, the principal at school level, and finally the participants and their parents. The study protocol was approved by Human Research Ethics Committee at the University of Newcastle and Ethical Committee in Medical Research at Xi'an Jiaotong University.

Height without shoes was measured using a non-stretchable tape suspended from the wall (214 Road RodTM, US) and was recorded to the nearest 0.1 cm. Body weight was measured without shoes but with underwear in the summer session, or light clothes in the autumn session, using a digital scale (Tanita HD-305, Japan) to the nearest 0.1 kg. Anthropometry was collected in schools by trained public health workers using anthropometric standardization reference manual [13]. Parents of the participants were asked to complete a household questionnaire in which the birth date of the participating children, parental education (in years) and self-reported height and weight were included. An inventory of household assets (house, TV, camera, DVD, air conditioner, transportation facilities, and kitchen utensils) was also recorded for computing a wealth index as an indicator of the socioeconomic status of the household [14].

Three groups of school environment factors were included in this study, namely school physical factors, curricula and policies. Physical factors included school size (enrolment), availability of sports facilities (playground measured as m^2 ^per student, oval, gym, and sports equipment), availability of canteen and selected foods and drinks (sweets and chocolates, ice-cream, soft drinks), and western fast food restaurants near school within 10-minute's walk (yes, no). School curricula covered schedules for sports meetings, morning exercises, class recess sports, and physical and health education lessons. School policies included bike riding (forbidding bicycle riding to school), snacking (allowing purchase of snacks at shops in the school), and foods sold at canteen (any regulations). These information was collected by asking school doctors to complete a pre-coded questionnaire (30 items) designed for this survey. Questionnaire items were identified based on three focus group discussions with the local school community stakeholders including staff from the Municipal Education Department, school health workers at district level, school teachers, students and their parents. Following the construction of the questionnaire, a scientific panel meeting at Xi'an Jiaotong University revised the questionnaire. The questionnaire was piloted at schools and further revised before the commencing the survey. Data was analyzed using the statistical package STATA version 10 (2005, STATA Corporation, College Station, TX, USA). Body mass index (BMI) was calculated by dividing body weight in kg by the square of height in meters. Overweight and obesity were defined using IOTF criteria [15]. BMI was used as the outcome variable in linear mixed effects models [16]. The fixed effect (coefficients) of school environment factors (selected from univariate models P < 0.25) with their 95% confidence intervals (95% CI) are presented in the results. The factors were adjusted for socio-demographic factors including age, parental education and their BMI, and household wealth. Univariate model was first conducted to screen variables with P < 0.25 for multivariate regression [17]. Significant socio-demographic factors were added with school factors in the subsequent models.

## Results

Of the 1804 students invited, 1792 (99.3%) students consented and had their weight and height measured. All of the 30 school doctors completed the school environment questionnaire. The household questionnaire was completed by 1768 parents of the participants.

Table 1 shows the characteristics of the participating schools, including student enrolments, age, gender distribution, and the prevalence of combined overweight/obesity. Socioeconomic factors such as parental education and their BMI, and household wealth, are also presented in Table 1. The school prevalence of overweight/obesity was the lowest in Baqiao district (10.1%) which is a semi-rural area, and was the highest in Yanta district (33.4%) in areas around city centre. Similar district pattern was found with parental education and their BMI, and household wealth. BMI was significantly associated with age (coef. 0.3, 95% CI: 0.1-0.4), parental education (coef. 0.8, 95% CI: 0.5-1.1), parental BMI (coef. 0.3, 95% CI: 0.2-0.4), and household wealth (coef. 0.3, 95% CI: 0.2-0.4), but not associated with gender (coef. -0.002, 95% CI: -0.3-0.3). Ten out of 20 school environment factors with a P < 0.25 in univariate analysis are shown in Table 2. BMI was negatively associated with play ground size, lack of school oval, and forbidding bicycle ride to school, but positively associated with lacking of morning exercise, or class recess exercise, or physical health education class, or less frequent sports-meeting, or the availability of soft drinks at school shops and western fast food outlets in school area. After adjusted for socio-demographic factors, the availability of soft drinks at school and western fast food in schools area were associated with BMI; school curricula such as sport meeting, health education sessions were associated with BMI.

**Table 1:** The school characteristics (n = 30)

School name	Enrolment	Age (SD)	%Female	Parental BMI	Parental education (years)	Household wealth ^*^	%Overweight/obesity ^**^
Xincheng District							

29th	258	13.8 (1.0)	54.1	22.1	9.4	-1.0	11.5
89th	3752	13.7 (1.0)	50.0	22.1	11.2	0.7	21.7
Aizhi	3095	13.7 (1.0)	51.7	22.9	13.5	1.9	16.7
Huanghe	2125	13.6 (1.0)	48.3	22.6	12.2	1.2	18.3
Xiguang	1857	13.7 (1.0)	49.2	22.7	13.1	1.0	15.3
Dahua	987	13.9 (0.9)	46.7	22.5	10.3	-0.7	10.0
Total	12074	13.7 (1.0)	50.0	22.5	11.6	0.5	15.6

Beilin District							

8th	2277	13.6 (1.0)	45.8	22.7	12.1	1.3	28.8
82th	820	14.6 (1.0)	52.5	23.2	10.9	0.3	11.5
Zunde	534	13.6 (1.0)	45.0	23.1	11.3	0.6	20.0
Jiankeda	663	14.2 (0.9)	50.9	22.7	13.4	1.3	15.3
Shenjian	593	14.6 (1.0)	47.3	23.1	10.9	0.0	12.7
Total	4887	14.1 (1.1)	48.3	23.0	11.7	0.7	17.7

Lianhu District							

15th	746	14.3 (1.1)	48.3	22.3	10.3	-1.1	11.7
67th	1040	14.2 (1.0)	48.3	22.1	9.5	-0.8	18.3
Dianli	1802	13.5 (1.0)	49.2	22.5	11.5	0.5	21.3
YD1st	2583	13.6 (1.0)	51.7	23.2	12.4	1.0	18.3
YD2nd	3744	13.8 (0.9)	61.7	22.6	10.8	0.3	20.0
Total	9915	13.9 (1.0)	51.8	22.6	10.9	0.01	17.9

Yanta District							

46th	282	14.2 (1.1)	48.3	23.2	8.7	-1.5	15.0
98th	1323	13.9 (1.0)	50.0	23.1	11.8	0.3	15.0
Gaoxin	2957	13.7 (1.0)	50.0	23.3	13.7	2.2	53.3
Dianzi	1206	13.8 (1.1)	40.0	22.7	10.8	0.0	21.7
Shida	1506	13.6 (1.0)	46.7	23.1	14.4	1.8	23.3
Total	7274	13.8 (1.0)	47.0	23.1	11.8	0.5	25.7

Weiyang District							

16th	1455	14.2 (1.0)	51.7	22.6	9.0	-0.5	10.0
50th	1638	14.0 (0.9)	47.5	22.7	8.7	-1.7	5.1
75th	1044	13.8 (1.0)	50.0	22.9	11.2	0.5	18.3
Shanshi	1036	13.9 (1.0)	56.9	22.6	9.7	-0.4	13.8
Qunxing	139	14.5 (1.4)	50.0	22.1	9.5	-0.4	6.7
Total	5312	14.1 (1.1)	51.2	22.6	9.6	-0.5	10.8

Baoqiao District							

55th	896	14.0 (1.0)	48.3	22.9	8.9	-1.8	10.1
64th	1318	14.1 (0.9)	50.0	22.1	9.7	-2.6	8.3
Luyuan	581	14.0 (0.9)	55.0	22.5	9.2	-2.2	6.7
Dianjian	878	13.7 (0.9)	50.0	22.7	10.6	0.1	15.0
Total	3673	13.9 (1.0)	50.8	22.5	9.6	-1.6	8.8

* Mean household wealth index per school, household wealth index computed by principal component analysis [14];

**Overweight and obesity was defined by IOTF BMI cut offs [15]

**Table 2:** BMI of and school environment factors in Xi'an City

School Factors		School No. or mean (SD)	**Student No**.	BMI (SD)	Unadjusted		Adjusted ^*^	
					Coef^**^	95% CI	Coef**	95% CI
**Playground per person (10 m**^**2**^**)**		0.5 (0.4)***	N/A	N/A	-0.5	-1.2, 0.3	-0.2	-0.9, 0.4
**School oval**	Yes	27	1619	20.0 (3.4)	Reference			
	No	3	173	19.3 (3.1)	-0.6	-1.6, 0.3	-0.6	-1.4, 0.2
**Morning exercise**	Yes	23	1379	19.9 (3.4)	Reference			
	No	7	413	20.1 (3.3)	0.3	-0.5, 1.0	0.1	-0.6, 0.7
**Class recess exercise**	Yes	28	1673	19.9 (3.4)	Reference			
	No	2	119	20.2 (2.9)	0.3	-1.0, 1.5	-0.2	-1.8, 0.9
**Sports meetings**	twice a year	13	779	19.7 (3.2)	Reference			
	Once a year	15	893	19.8 (3.3)	0.1	-0.4, 0.6	0.1	-0.3, 1.6
	Once in 2 years	2	120	22.0 (4.0)	2.2	1.2, 3.2	1.9	1.0, 2.8
**Health education**	Yes	27	1614	19.8 (3.3)	Reference			
	No	3	178	21.4 (3.9)	1.6	0.7, 2.4	1.2	0.4, 2.0
**Soft drinks at school shops**	No	2	181	19.8 (3.2)	Reference			
	yes	8	1619	21.2 (4.1)	1.4	0.5, 2.3	1.2	0.4, 2.0
**Fast food outlets**	No	21	1259	19.7 (3.8)	Reference			
	Yes	9	541	20.5 (3.1)	0.8	0.2, 1.5	0.7	0.1, 1.2
**Number Fast food outlets**	0	16	954	19.2 (3.0)	Reference			
	1	8	477	20.3 (3.5)	0.8	0.1, 1.4	0.6	-.0.02, 1.1
	≥2	6	361	20.5 (3.9)	1.0	0.3, 1.7	0.8	0.1, 1.4
**Bike riding (allowed)**	Yes	29	1740	20.0 (3.4)	Reference			
	No	1	60	18.7 (2.4)	-1.2	-2.9, 0.4	-0.8	-2.2, 0.6

* Adjusted for significant socio-demographic factors including age, parental education, parental BMI, and household wealth Index.

**coefficient of school factors was from linear mixed effect models

*** mean playground per person in 10 m^2 ^with the standard deviation.

## Discussion

This study indicated the school environment factors such as availability of soft drinks at school and fast food outlet in school area, and school curricula were associated with BMI independent of socio-demographic factors. And number of fast food outlet appeared to be dose responsive with BMI. These results are useful to generate future research hypothesis and to plan obesity prevention programs that include environment components. One future research could be on how availability of soft drinks and fast food outlet influence the dietary habit of students. Another direction could be to evaluate the effect of school curricula modification on individual healthy behaviours such as physical activity, and knowledge about health diet.

The association between fast food outlets around school area and BMI found in this study was consistent with the US studies [8,18], although the effect size varied due to methodological differences in outcome variable and statistical analysis. Children who ate fast food consumed more total energy, more total fat, less fibre, and fewer fruits and non starchy vegetables [19]. Frequent consumption of western fast food was associated with overweight and obesity in Chinese boys [20]. Fast food restaurant use has increased rapidly in urban areas in China and has become popular among children and adolescents massive advertising and marketing campaigns. For example, KFC had 10 restaurants in 1992 and 2500 in 2009, while McDonald's will double its number of outlets in China in the next three or four years to 2,000 by 2015 [21]. Adolescents living in urban areas are socioeconomically better in the present study and are exposed to the obesity-promoting food environment. It is becoming crucial to equip them with healthy diet knowledge and to inform community authorities of the evidence for future city planning to avoid the exposure. This is contrary to the results from recent studies conducted in Canada and New Zealand using GIS that higher density of fast-food outlets in more-socially deprived school areas [22,23]. US studies indicated it is the lower price but not the availability of fast food that was associated with BMI [24].

Consumption of soft drinks has been established to be associated with overweight and obesity in adolescents both in western society and developing countries from longitudinal [25] and cross-sectional studies [20,26,27]. The exposure of soft drinks at schools and lack of parents' supervision may increase the consumption of soft drink. A US representative data revealed that sugar-sweetened beverages obtained at school contributed a daily mean of 29 kcal in middle school children and 46 kcal in high school children [28]. The study also showed that attending a school without stores or snack bars was estimated to reduce sugar-sweetened beverage consumption by 22 kcal per school day in middle school children and by 28 kcal in high school children [28]. Chinese health authorities proposed to ban soft drinks at schools following the practice in US, UK, German, and Japan [29].

School curricula such as less sports-meeting and no health education class were associated with higher BMI in Chinese students. Chinese students are under academic pressure due to the examination system and spend most of their waking time attending classes and doing homework [30]. The health authorities and relevant government agencies have decided to tackle the declining health of young people including overweight and obesity in schools, for example, in 2006, the Chinese Ministry of Education launched the Sunshine Sports program to promote physical activity in schools [31]. The aim of the program was to help 85% school students achieving one hour physical activity and 9-10 hours of sleep within three years for improving their health [31]. Following a success multicenter school intervention project in China to promote physical activity and health knowledge among primary students [32], Beijing Municipal Education Bureau has launched a campaign of "5 more minutes class recess exercise" [33]. Schools are required to reform the curricula to have physical education and health education session twice a week instead of once a week. The ultimate goal is to have student do at least one hour of physical activity a day in school [33]. Practical school health policies and curricula will be developed and implemented after evaluation of these programs [31-33].

Compared with our previous hierarchical logistic regression model in which factors at the levels of community, school, household, parental and students' individual were studied [10], different school factors were identified. This is not contradictory since each analytic approach aimed to answer different question. The purpose of this analysis was to examine the association between school environment factors and BMI adjusted for socio-demographic factors; while the hierarchical analysis explored the associated risk factors in the presence of all inter-related factors at different levels. The interpretation and understand of the results of the two analysis should be scrutinized.

This study enrolled a representative large sample with high response rate. The outcome variable was collected using standardized objective measurements. In addition, the statistical method took into account both within and between school variations of the school factors. The strength of this study contributed robust and unbiased effect size estimation. Schools in China generally adopt uniform system in management, structure and curricula, so the results from this study are applicable to other schools in China and it serves the first step in understanding the relationship between school environment and BMI to generate future research. In spite of these, the cross-sectional study could not provided causal relationship between school environment factors and weight status. Other factors that associate with BMI at household and community level influencing individual behaviours are not reported in this study.

## Conclusions

School environment factors such as the availability of soft drinks at school, easy access to fast food outlet in school area, and school curricula were associated with students' BMI in Xi'an City, China. Community and school policy makers should make efforts for students to avoid exposure to fast food outlet in school area and soft drinks at school shops, and to improve school curricula to promote sustainable healthy lifestyle behaviours.

## Competing interests

The authors declare that they have no competing interests.

## Authors' contributions

ML contributed to study design, project management, data collection and analysis, manuscript preparation. MJD participated study design, supervising data collection and manuscript revision. YH involved in supervision of data collection and project management. All authors read and approved the final manuscript.

## Pre-publication history

The pre-publication history for this paper can be accessed here:

http://www.biomedcentral.com/1471-2458/11/792/prepub
